# In Memoriam: Thomas J. Gryczan (1949–2024)

**DOI:** 10.3201/eid3009.240736

**Published:** 2024-09

**Authors:** Byron Breedlove, Barbara Segal

**Keywords:** In Memoriam, Thomas J. Gryczan

Thomas J. Gryczan, better known to his coworkers and many friends as Tom, worked from May 2005 until his death in March 2024 with the Centers for Disease Control and Prevention as a dedicated, highly focused technical writer-editor for *Emerging Infectious Diseases* (EID). During his tenure with the journal, Tom was recognized for his unwavering enthusiasm for work, willingness to share knowledge about science and publishing, and ability to handle challenging assignments and tight deadlines. Tom contributed to EID in many other ways, including mentoring new staff and helping coordinate and administer continuing medical education credits offered by the journal.

EID editor-in-chief Peter Drotman recalled Tom’s knowledge was almost boundless. “One of the many places where he put his skills to good use was in assisting authors of the popular Etymologia entries in crafting their historical vignettes,” Drotman said. “Tom edited more than 100 of those articles, all of which are uncredited. Tom was an ardent supporter of the goal of this EID feature, which is to enhance the knowledge of students of microbiology and infectious diseases about the heritage of our field and how the names we use came to be.”

Tom held a bachelor’s degree from Marist College in New York, a master’s degree from Long Island University, and a PhD in microbiology from New York University. Before joining CDC, Tom worked as a researcher at the Public Health Research Institute, International Center for Public Health, at New Jersey Medical School at Rutgers University. Throughout his career, he authored 16 scholarly articles that have been cited 1,168 times to date.

In the mid-1980s, Tom embarked on a nearly four-decade career as a technical writer-editor, initially with Gordon & Breach Science Publishers in New York. After relocating to Atlanta, Georgia, he worked for the *American Journal of Tropical Medicine and Hygiene *and* Arthritis & Rheumatology*. He also worked as an editor for the American Cancer Society.

Tom’s puns and bad jokes brought levity to many EID staff meetings, and he often delighted colleagues with homemade treats from his kitchen during EID’s weekly gatherings, before the COVID-19 pandemic required social distancing. Beyond his professional pursuits, he had diverse interests, including gardening, bowling, roller skating, listening to disco music, cooking, reading nonfiction, studying history, and watching hockey and baseball.

Tom was a born teacher and was knowledgeable in countless subject areas. One of Tom’s daughters noted that “Tom was Google before there was a Google.” For instance, he could list every World Series winner from the 1950s to 2023 and how many games were played in each series. He could also provide details on all the elements in the periodic table or recount historical events, including precise dates and locations. Colleagues recall many enthusiastic breakroom conversations with Tom on myriad subjects that invariably ended with learning new facts and insights. If someone mentioned a trivia interest, it then became Tom’s thoroughly researched passionate interest as well. Despite his enthusiasm and extroverted personality, Tom was notably modest except when talking about family, and then he could not contain his pride.

We offer our condolences to Tom’s surviving family members, including his wife, daughters, grandsons, and sisters, and to his many friends and acquaintances at CDC and around the world.

**Figure:**
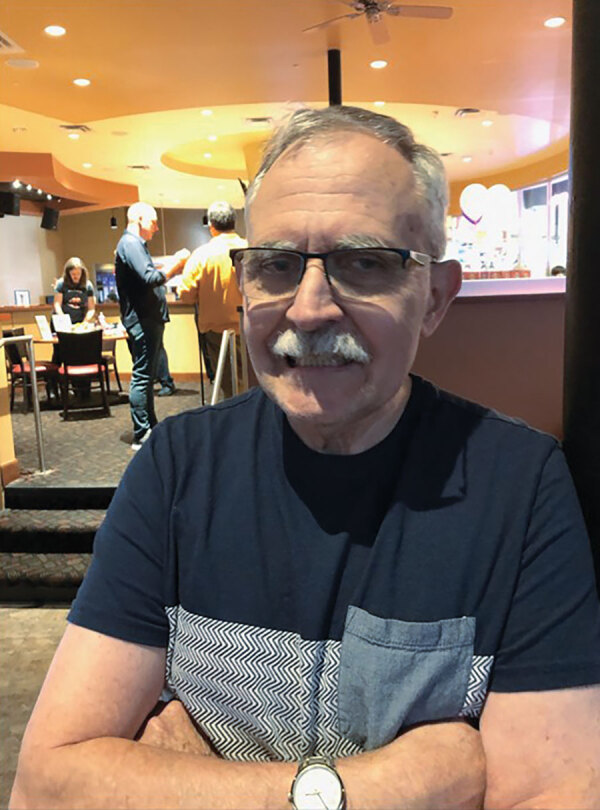
Thomas J. Gryczan. Photo credit: Susan Twadell.

